# Supplementary material for: Amyloid-Forming Corpora Amylacea and Spheroid-Type Amyloid Deposition: Comprehensive Analysis Using Immunohistochemistry, Proteomics, and a Literature Review
Source: Int J Mol Sci. 2024 Apr 4;25(7):4040. doi: 10.3390/ijms25074040 (PMC11012059; doi:10.3390/ijms25074040)
Supplement: Supplementary file 1 [file ijms-25-04040-s001.zip › ijms-2927064-supplementary.pdf]

Online Supplemental Material

Supplementary Table S1. Details of the antibodies used in this study.

Antibody	Source	Clone	Dilution	Antigen retrieval
Igλ	Abcam	polyclonal	1:1600	98% FA (1 min)
β2-microglobulin	GeneTex	polyclonal	1:3000	98% FA (1 min)
Lysozyme C	Santa Cruz Biotechnology	E-5	1:150	98% FA (1 min)
PSP-A	Abcam	6F10	1:4000	98% FA (1 min)
PSP-B	Santa Cruz Biotechnology	F-2	1:4000	98% FA (1 min)
Lactoferrin	Santa Cruz Biotechnology	B97	1:150	98% FA (1 min)
Olfactomedin 4	Abcam	polyclonal	1:50	Heat (20 min, pH6)
p62	Biomol	polyclonal	1:3000	Heat (20 min, pH6)

Abbreviations: FA, formic acid; PSP-A, pulmonary surfactant protein A; PSP-B, pulmonary surfactant protein B; Immunostaining was performed using the Leica Bond-IV automation and Leica Refine detection kits (Leica Biosystems, Bannockburn, IL, USA) according to the manufacturer's instructions. All sections were counterstained with hematoxylin.

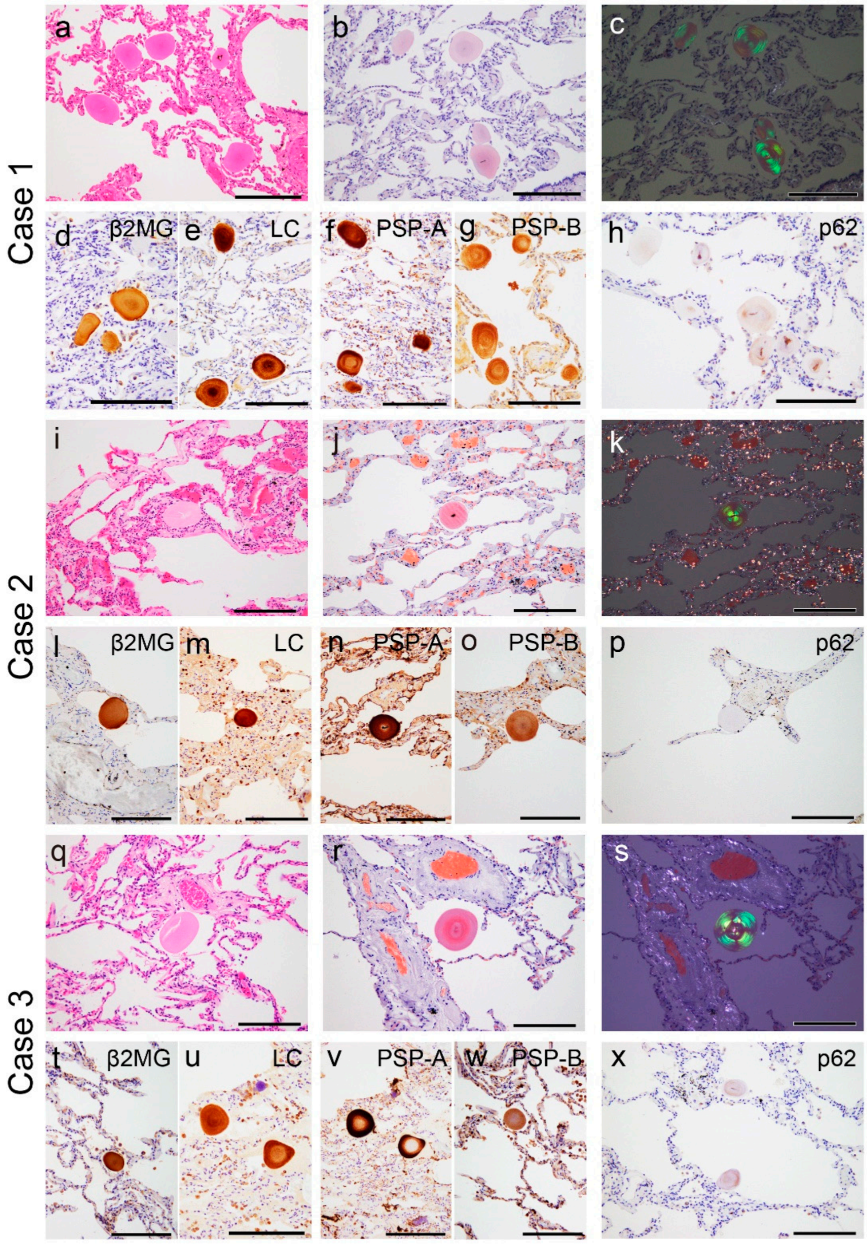

Supplementary Figure S1. Representative microphotographs of the pulmonary corpora amylacea (CA) in all three cases. (a–h) Pulmonary CA Case 1; (i–p) Case 2; (q–x) Case 3. (a, i, q) Hematoxylin and eosin (H&E) staining; phenol Congo red (pCR) staining under bright field (b, j, r) and polarized light (c, k, s); Immunohistochemistry for β2-microglobulin (β2MG) (d, l, t); lysozyme C (LC) (e, m, u); pulmonary surfactant protein A (PSP-A) (f, n, v); PSP-B (g, o, w); and p62 (h, p, x). In all cases, CAs were round and eosinophilic deposits showed concentric laminations by H&E staining. In addition, all CAs exhibited congophilic and clear apple-green birefringence under polarized light. Furthermore, all CAs showed immunoreactivity for β2MG, LC, PAP-A, and PSP-B. In contrast, immunoreactivity for p62 was weak and focal in Cases 1 and 3, whereas it was almost negative in Case 2. Scale bar = 200 µm (a–x).

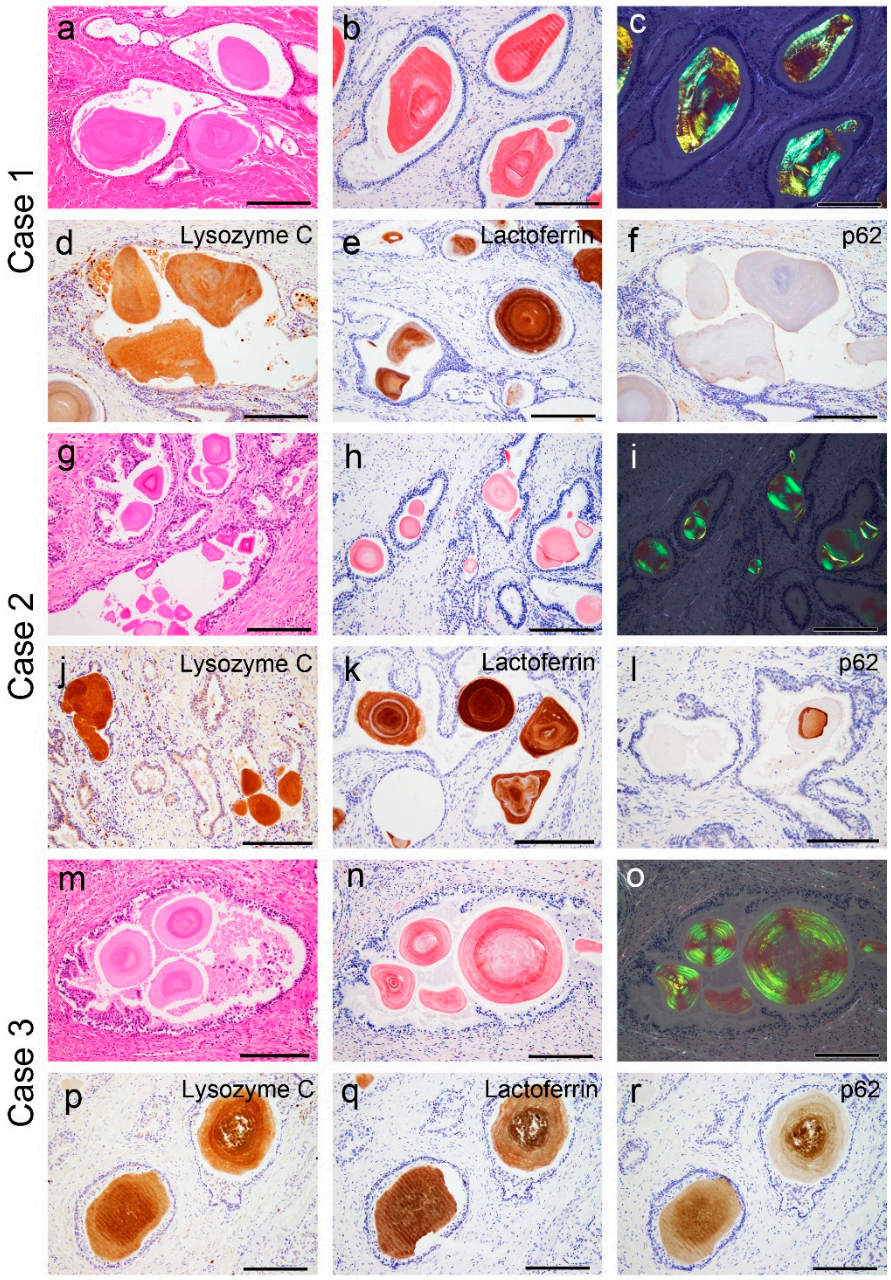

Supplementary Figure S2. Representative microphotographs of prostatic CA in all three patients. (a–f) Prostatic CA Case 1; (g–l) Case 2; and (m–r) Case 3. (a, g, m) H&E staining; pCR staining under bright field (b, h, n) and polarized light (c, i, o); Immunohistochemistry for lysozyme C (d, j, p); lactoferrin (e, k, q); and p62 (f, l, r). For all cases, the CAs were round and eosinophilic deposits showed concentric laminations by H&E staining. In addition, all CAs exhibited congophilic and clear apple-green birefringence under polarized light. Furthermore, all CAs showed immunoreactivity for lysozyme C and lactoferrin. Immunoreactivity for p62 was moderate in Case 3, but weak and focal in Cases 1 and 2. Scale bar = 200 µm (a–r).

a PSP-A

1 MWLCPLALTL ILMAASGAAC EVKDVCVGSP GIPGTPGSHG LPGRDGRDGV

51 KGDPGPPGPM GPPGETPCPP GNNGLPGAPG VPGERGEKGE AGERGPPGLP

123-137 138-157

101 AHLDEELQAT LHDFRHQILQ TRGALSLQGS IMTVGEKVFS SNGQSITFDA

167-179 181-199

151 IQEACARAGG RIAVPRNPEE NEAIASFVKK YNTYAYVGLT EGPSPGDFRY

201 SDGTPVNYTN WYRGEPAGRG KEQCVEMYTD GQWNDRNCLY SRLTICEF

b PSP-B

1 MAESHLLQWL LLLLPTLCGP GTAAWTTSSL ACAQGPEFWC QSLEQALQCR

95-106

51 ALGHCLQEVW GHVGADDLCQ ECEDIVHILN KMAKEAIFQD TMRKFLEQEC

101 NVLPLKLLMP QCNQVLDDYF PLVIDYFQNQ TDSNGICMHL GLCKSRQPEP

201-212

151 EQEPGMSDPL PKPLRDPLPD PLLDKLVLPV LPGALQARPG PHTQDLSEQQ

218-224 225-252

201 FPIPLPYCWL CRALIKRIQA MIPKGALAVA VAQVCRVVPL VAGGICQCLA

253-264 265-272 287-295

251 ERYSVILLDT LLGRMLPQLV CRLVLRCSMD DSAGPRSPTG EWLPRDSECH

337-352

301 LCMSVTTQAG NSSEQAIPQA MLQACVGSWL DREKCKQFVE QHTPQLLTLV

351 PRGWDAHTTC QALGVCGTMS SPLQCIHSPD L

Supplementary Figure S3. Proteomic analysis results for PSP-A and PSP-B in pulmonary CA lesions in Case 1. (a) PSP-A and (b) PSP-B. Detected peptides with peptide scores of 30 or higher in the MASCOT analysis are shown in red.

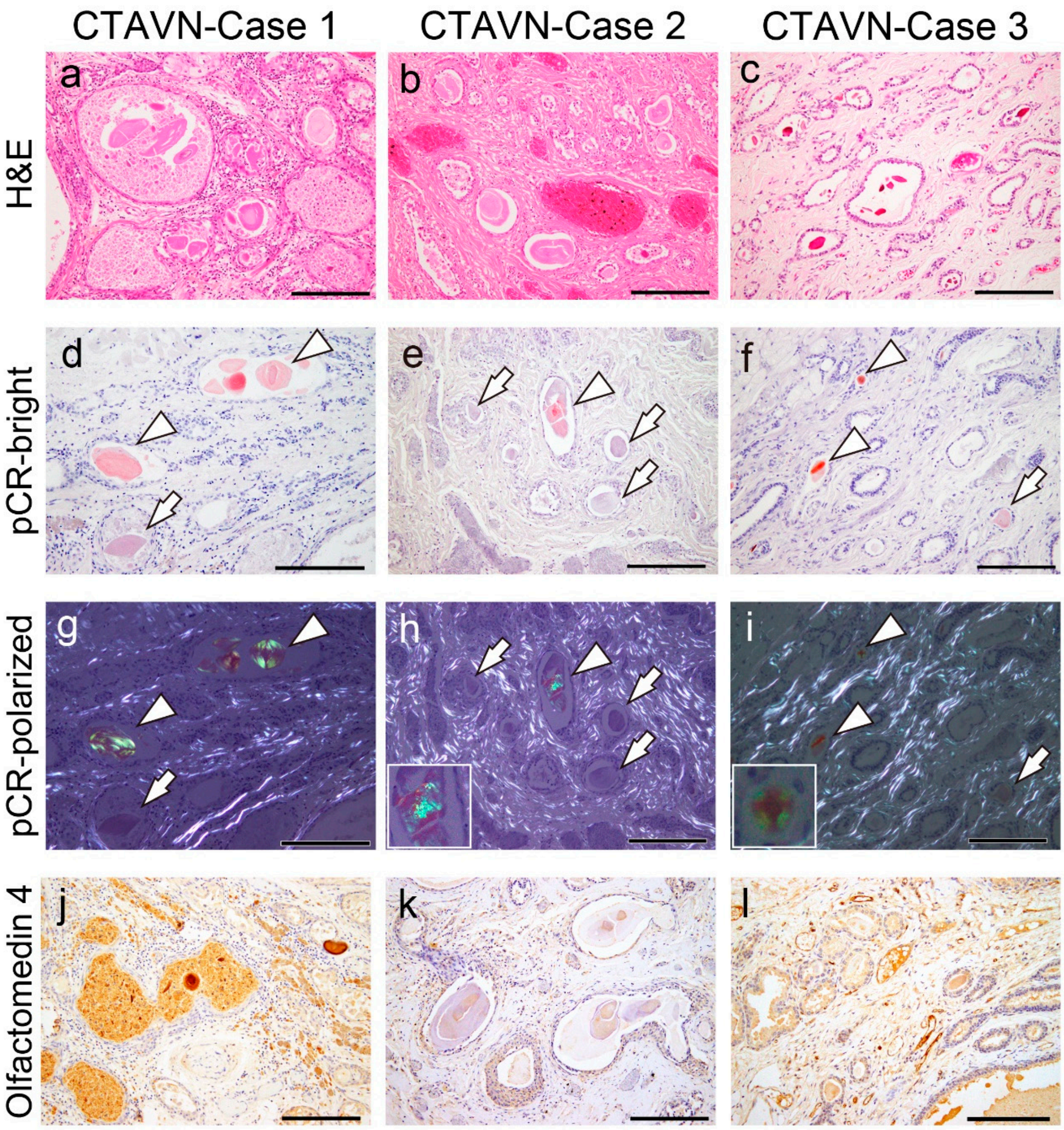

Supplementary Figure S4. Representative microphotographs of cystic tumors of the atrioventricular node (CTAVN)-associated CA in all three cases. (a, d, g, j) Prostatic CA Case 1; (b, e, h, k) Case 2; (c, f, i, l) Case 3. (a–c) H&E staining; pCR staining under bright field (d–f) and polarized light (g–i); (j–l) Immunohistochemistry for olfactomedin 4. For all cases, the CAs were round and eosinophilic deposits showed concentric laminations by H&E staining. For pCR staining, the CAs showed very weak (arrow) to moderate congophilia (arrowhead) (d–f), and those exhibiting moderate congophilia showed apple-green birefringence under polarized light (arrowhead) (g–i). Immunoreactivity of the CAs to olfactomedin 4 was strong in Case 1 (j), negative to weakly positive in Case 2 (k), and weakly positive in Case 3 (l). Scale bar = 200 µm (c–e).
